# Editorial: Cities and mental health

**DOI:** 10.3389/fpsyt.2023.1263305

**Published:** 2023-08-04

**Authors:** Agnieszka Olszewska-Guizzo, Alessio Russo, Adam Charles Roberts, Simone Kühn, Bruno Marques, Nour Tawil, Roger C. Ho

**Affiliations:** ^1^NeuroLandscape Foundation, Warsaw, Poland; ^2^School of Arts, University of Gloucestershire, Cheltenham, United Kingdom; ^3^Future Resilient Systems, Singapore ETH Centre, Singapore, Singapore; ^4^Lise Meitner Group for Environmental Neuroscience, Max Planck Institute for Human Development, Berlin, Germany; ^5^Clinic and Policlinic for Psychiatry and Psychotherapy, University Medical Center Hamburg-Eppendorf, Hamburg, Germany; ^6^Faculty of Architecture and Design Innovation, Victoria University of Wellington, Wellington, New Zealand; ^7^Department of Psychological Medicine, National University of Singapore, Singapore, Singapore

**Keywords:** cities, urban, mental health, wellbeing, greenspace, urban planning

The link between urbanization and mental health is multifaceted and draws considerable attention from researchers and professionals worldwide. The Research Topic “Cities and Mental Health” dives into this theme, illuminating the influences of urban living on mental health and some possible causal pathways. Specifically, this editorial investigates the complexity of the relationship between urbanization and mental health, highlighting the increased interest among scholars and practitioners around the world. Here we present an overview of current studies by synthesizing twelve research articles published in the Research Topic. The majority of these studies are centered on China, followed by two studies from Germany, two from Taiwan and one from Portugal. According to the word analysis conducted with Nvivo 12 Pro software that visualizes the 1,000 most frequent words retrieved from the published articles, terms: “social” “health” and “urban” are the most frequently used by the authors. In Figure 1 we show the results of the word analysis in a form of a word-cloud.

**Figure 1:**
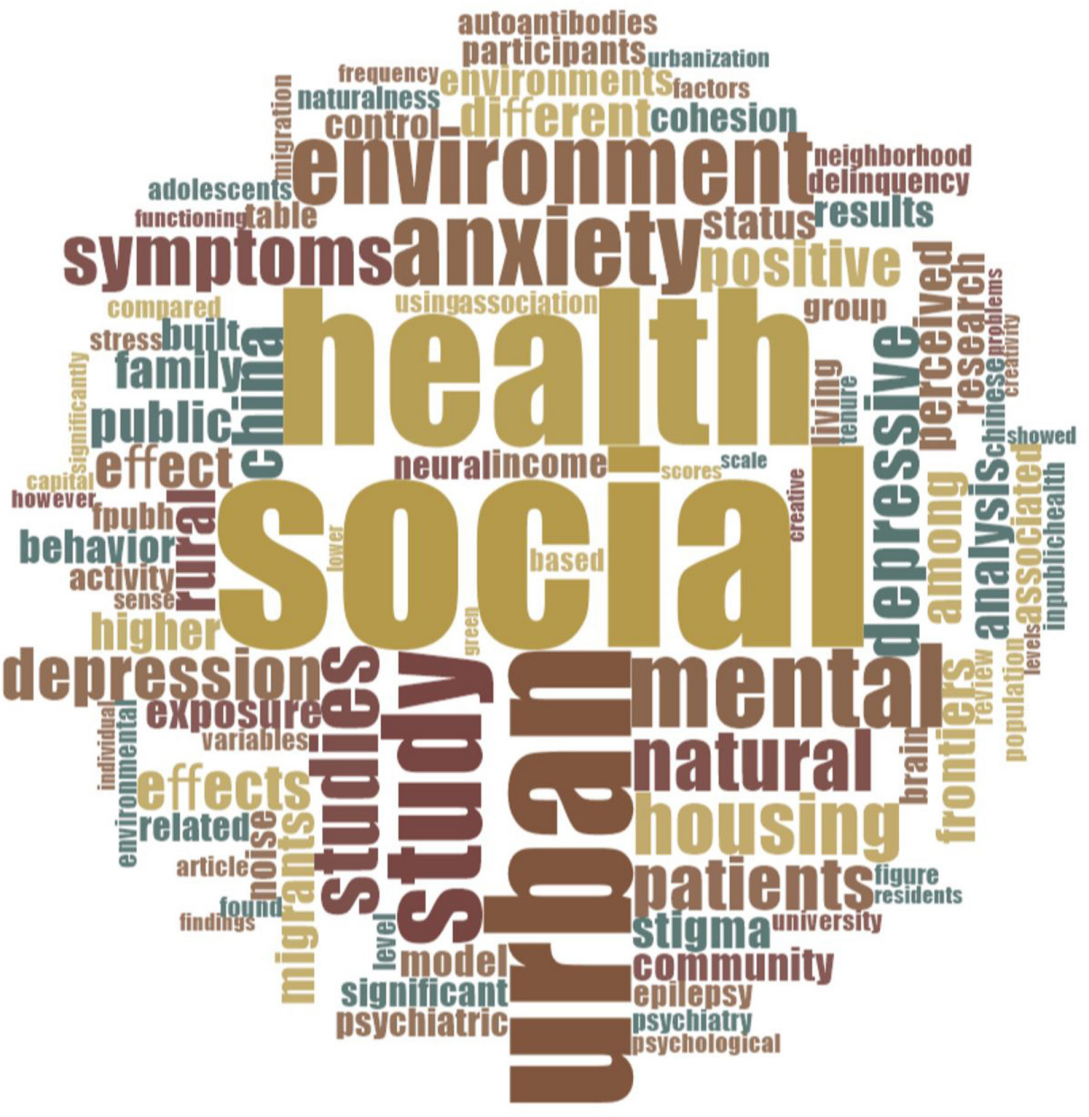
Word cloud generated by Nvivo 12 Pro illustrating the 1,000 most frequent words extracted from the analysis of 12 articles. The terms “social” and “health” dominate the center of the image, indicating their significant prevalence within the dataset.

The social aspects of urban life emerge as critical determinants of mental health. One study in the collection associates perceived social cohesion with depressive symptoms among internal migrants in China, with social adaptation acting as a mediator (Qu et al.). In it, authors demonstrate that fostering social cohesion and promoting social strategies can help alleviate the mental health disparities among this vulnerable population, as well as highlighting the especially vulnerable group of involuntary rural-to-urban migrants, who may require additional social support. Furthermore, the impact of housing tenure mix on mental health is examined in the megacity of Guangzhou, China, revealing that while there is no direct impact, social participation mediates the decrease in depressive symptoms (Zhang et al.).

Moving into family dynamics, a paper from Changsha, the capital of Hunan province, reports increasing trends of various mental health problems among Chinese adolescents in urban areas between 2016 and 2020, emphasizing the urgency to address mental health issues in cities, especially among younger demographics (Wu et al.). Another article explores the protective role of good family functioning against adolescent delinquency in another Chinese megacity (Wan et al.). The authors highlight the value of positive behavior recognition in family environments, which is relevant in both urban and rural settings.

Socioeconomic aspects in urban settings also show significant influence on mental health through the issue of perceived stigma as presented by Hohmann et al. in a German study. In it, the authors imply a need for targeted interventions and public education programs that take into account the varying social environments in urban areas to reduce stigma.

A contribution from Ancora and colleagues titled “Ci*ties and neuroscience research: a systematic literature review*” helps establish the broader background by investigating the neural mechanisms underlying cognitive and emotional processes in relation to urban environment exposure (Ancora et al.). The paper recommends policy-making for healthier and sustainable cities, emphasizing the considerable mental benefits of green spaces and urban upbringing with high nature exposure as well as the role of air pollution in neurodegenerative process and cognitive decline.

Further down this path, the influence of city environments on psychiatric disease development is tackled by a German study that explores the prevalence of neural autoantibodies in urban vs. rural psychiatric patients (Hansen et al.). The paper concludes that, while there were no significant findings in antibodies levels between rural and urban patients, more extensive studies are needed to explore the possible higher prevalence of autoimmune-related psychiatric syndromes in larger cities.

The urban soundscape's influence on mental health is broached by a Taiwanese study, reporting on significant associations between road traffic noise at specific frequencies and increased depression prevalence, even when controlling for the effects of pollution (Lin et al.). In another Taiwanese study the effects of natural environments on mental health are brought to light in a context of creativity in a study titled “The influence of natural environments on creativity” (Yeh et al.). The authors argue that viewing natural environments fosters flexibility and imagination, highlighting another dimension of how urban greening can influence productivity and wellbeing.

Following the research on sensory stimuli influencing the mental health Chinese meta-analysis by Tan and colleagues summarized the existing clinical trials on the essential oils in treatment of psychiatric disorders (Tan et al.). It concludes that natural scents of lemon and lavender showed to be the most effective in treatment of anxiety.

An important aspect touched upon by this Research Topic is the mental health of urban professionals and their productivity, with two papers investigating these issues. One paper documents higher depression prevalence among medical staff in Hainan, China, associating it with anxiety, sleep disorders, and job-related stressors (Lu et al.). In contrast, another study reveals that planned urbanization can improve mental health among middle-aged and older adults in China by promoting social participation and improving living conditions (Hong et al.). This dichotomy underscores the nuanced impacts of urban life on mental wellbeing, depending on one's occupation and socioeconomic status.

In conclusion, these papers collectively underscore the significant interplay between urban environments and mental health. The findings affirm the imperative to consider multifaceted factors including environmental, social, and economic aspects in urban planning and health policy-making, and as a whole show commonalities across urban areas in different countries. While a lot of research is yet to be done to identify positive interventions, the effect of natural environments, multi-sensory exposure, and participatory approaches at the neighborhood levels seem to be the most promising tools to alleviate the burden of mental health in cities while promoting productivity and creativity. This collection of studies prompts researchers and policymakers to discuss and work together toward healthier, greener and more supportive cities for better mental health outcomes.

## Author contributions

AO-G: Conceptualization, Writing—original draft, Writing—review and editing. AR: Visualization, Writing—review and editing. ACR: Writing—review and editing. SK: Writing—review and editing. BM: Writing—review and editing. NT: Writing—review and editing. RH: Writing—review and editing.

